# Polygenic Risk Score Predicts Prostate Cancer Risk Independent of Type 2 Diabetes

**DOI:** 10.1111/dom.70690

**Published:** 2026-03-26

**Authors:** Guk Jin Lee, Sang‐Hyuk Jung, Jonghyun Lee, Ki Won Moon, Penn Medicine Biobank, Daniel J. Rader, Marylyn D. Ritchie, JoEllen Weaver, Nawar Naseer, Giorgio Sirugo, Afiya Poindexter, Yi‐An Ko, Kyle P. Nerz, Meghan Livingstone, Fred Vadivieso, Stephanie DerOhannessian, Teo Tran, Julia Stephanowski, Salma Santos, Ned Haubein, Joseph Dunn, Colleen Morse Kripke, Marjorie Risman, Renae Judy, Colin Wollack, Anurag Verma, Shefali S. Verma, Scott Damrauer, Yuki Bradford, Scott Dudek, Theodore Drivas, Jae‐Seung Yun, Dokyoon Kim

**Affiliations:** ^1^ Division of Medical Oncology, Department of Internal Medicine, Bucheon St. Mary's Hospital, College of Medicine The Catholic University of Korea Seoul Republic of Korea; ^2^ Department of Biostatistics, Epidemiology and Informatics, Perelman School of Medicine University of Pennsylvania Philadelphia Pennsylvania USA; ^3^ Department of Medical Informatics Kangwon National University College of Medicine Chuncheon Republic of Korea; ^4^ Interdisciplinary Graduate Program in Medical Bigdata Convergence Kangwon National University Chuncheon Republic of Korea; ^5^ Division of Rheumatology, Department of Internal Medicine Kangwon National University College of Medicine Chuncheon Republic of Korea; ^6^ Institute for Translational Medicine and Therapeutics, Perelman School of Medicine University of Pennsylvania Philadelphia Pennsylvania USA; ^7^ Division of Endocrinology and Metabolism, Department of Internal Medicine, St. Vincent's Hospital, College of Medicine The Catholic University of Korea Seoul Republic of Korea; ^8^ Institute for Biomedical Informatics University of Pennsylvania Philadelphia Pennsylvania USA

**Keywords:** diabetes mellitus, insulin‐like growth factor‐1, polygenic risk score, prostatic neoplasms

## Abstract

**Aims:**

Type 2 diabetes mellitus (T2DM) has been inversely associated with prostate cancer (PrCa) risk. However, it remains unclear whether a polygenic risk score (PRS) for PrCa can effectively stratify risk among men with T2DM. The primary objective of this study was to assess whether a PrCa PRS predicts PrCa risk independently of T2DM status. The secondary objective was to evaluate potential mediating factors, including insulin‐like growth factor‐1 (IGF‐1) and sex hormones.

**Materials and Methods:**

We analysed data from over 140 000 men in the UK Biobank and Penn Medicine Biobank. A PrCa PRS was constructed using summary statistics from a large‐scale genome‐wide association study. Cox proportional hazards models were used to evaluate the association between PRS and incident PrCa, adjusting for relevant covariates and testing for interaction by T2DM status. Additionally, sex hormones and IGF‐1 levels were analysed to explore potential mediators.

**Results:**

T2DM was associated with a reduced incidence of PrCa. The PrCa PRS was significantly associated with PrCa risk regardless of T2DM status (*p* < 0.001), and men in the highest PRS category exhibited the greatest risk, especially among those without T2DM. IGF‐1 levels were positively associated with PrCa risk among both diabetic and non‐diabetic men, while sex hormone levels showed no significant association in men with T2DM. Adjusting for testosterone and IGF‐1 did not attenuate the association between PRS and PrCa.

**Conclusions:**

PrCa PRS effectively stratifies risk among men with and without T2DM, highlighting the independent contribution of genetic susceptibility. Lower IGF‐1 levels in T2DM patients may partly mediate the reduced PrCa risk, suggesting a possible biological mechanism underlying these observations.

## 1 Introduction

Type 2 diabetes mellitus (T2DM) presents a clinical paradox in prostate cancer (PrCa). While diabetes has been associated with increased risk for several malignancies, including liver, pancreatic, and endometrial cancers, multiple epidemiologic studies and meta‐analyses have consistently reported a reduced incidence of PrCa among men with T2DM [1, 2, 3, 4]. Despite this apparent protective association, outcomes among diabetic men who develop PrCa are often worse [5, 6]. A meta‐analysis of cohort studies demonstrated that diabetes significantly increases both all‐cause and cancer–specific mortality among men with PrCa [7]. These findings create a practical clinical dilemma: although T2DM appears to lower PrCa incidence, clinicians still need to identify which men, particularly those with diabetes, remain at high risk and may benefit from targeted screening or early intervention.

PrCa is one of the most heritable common cancers, with genetic factors accounting for more than half of disease susceptibility in twin studies [8, 9]. Given this strong genetic contribution, polygenic risk scores (PRS), which aggregate the effects of numerous germline risk variants, have emerged as powerful tools for risk stratification of PrCa. However, it remains unclear whether genetic risk stratification performs similarly in individuals with T2DM, a population in whom overall incidence of PrCa appears reduced but prognosis may be poorer. Determining whether PRS retains predictive utility regardless of diabetes status is therefore essential for translating genetic risk assessment into real‐world clinical practice.

Several biological mechanisms have been proposed to explain the inverse association between T2DM and PrCa [10]. Men with T2DM often exhibit lower circulating testosterone levels, which may influence prostate carcinogenesis [11, 12, 13]. Additionally, relative hypoinsulinaemia and altered insulin‐like growth factor 1 (IGF‐1) signalling have been implicated in modulating PrCa cell proliferation [14, 15]. These metabolic and hormonal alterations raise the possibility that the relationship between T2DM and PrCa risk may be mediated, at least in part, by endocrine factors. Evaluating sex hormones and IGF‐1 alongside genetic susceptibility may therefore provide insight into how metabolic and inherited risks interact.

In this study, we aimed to address this clinical and biological gap by evaluating whether a comprehensive PrCa PRS stratifies PrCa risk independently of T2DM status. Using two large biobank cohorts, we examined the association between PRS and incident PrCa in men with and without T2DM and assessed the joint effects of genetic risk and diabetes status. As a secondary objective, we investigated whether circulating sex hormones and IGF‐1 were associated with PrCa risk and whether they might contribute to the observed inverse association between T2DM and PrCa. By integrating genetic and metabolic perspectives, this study seeks to inform more precise risk stratification strategies for PrCa in men with diabetes.

## 2 Materials and Methods

### 2.1 Study Population

The UK Biobank (UKBB) is a large prospective observational cohort study that has recruited > 500 000 adults across 22 centers located throughout the United Kingdom. The full protocol of the UKBB study is publicly available. Its study design and measurement methods have been described elsewhere [16]. Participants aged 40–69 years were enrolled between 2006 and 2010. Follow‐up began at baseline assessment (median~12.2 years to censoring/death linkage; minimum ≥ 1 year follow‐up). They were followed up for subsequent health events. For this study, we included only male individuals without prevalent prostate cancer at baseline who were diagnosed with International Classification of Diseases (ICD)‐9 or ‐10 codes or identified from hospital episode statistics.

The Penn Medicine Biobank (PMBB) served as an independent replication cohort to validate UKBB findings in a distinct population with electronic health record (EHR)‐based phenotyping (Figure S1). The PMBB is a large academic medical biobank that recruits participants from outpatient settings. All participants provided informed consent for access to their longitudinal EHR data starting from their first clinical encounter (defined as the PMBB EHR start time point; median~2010, range 2003–2019) and the generation of genomic and biomarker data [17]. This provided median~7.0 years of EHR coverage per participant (4.2 years post‐enrolment follow‐up; minimum ≥ 1 year follow‐up). ICD‐9 and ICD‐10 diagnosis codes, clinical imaging and laboratory measurements were extracted from EHRs up to July 2020. For this study, we focused on male participants with at least one recorded medical history and available genetic data.

### 2.2 Definition of Prevalent Type 2 Diabetes Mellitus

Participants with T2DM at baseline were identified by integrating multiple data sources, harmonized across UKBB (self‐reports, touch‐screen questionnaire, verbal interviews, and linked records) and PMBB (EHRs/lab data). Baseline T2DM was defined as ICD‐9 codes 250.00, 250.10, 250.20 and 250.90 or ICD‐10 codes E11 and E14, or lab criteria random glucose level ≥ 11.1 mmol/L or a glycated haemoglobin (HbA1c) level ≥ 48 mmol/mol (6.5%) according to the American Diabetes Association criteria [18]. We excluded individuals with diagnosis age < 18 years or type 1 DM by ICD‐10 code E10. This harmonized approach ensured comparable T2DM ascertainment between UKBB and PMBB.

### 2.3 Definition of Incident Prostate Cancer

Incident PrCa was defined as ICD‐9 code 185 or ICD‐10 code C61 derived from hospital inpatient records or, cancer and death registries [19, 20]. The endpoint was defined as the first diagnosis of PrCa, or death from PrCa, whichever was recorded first. Male participants with prevalent cancer were excluded from this study.

### 2.4 Biochemical Measurements and Covariates

Basic biochemical measurements and covariate data were obtained following established protocols. Detailed information on laboratory assays and data collection is described in Methods S2, S3 [21, 22, 23].

### 2.5 Genotype Data Quality Control and Imputation

Genotyping and quality control (QC) procedures and imputation followed standard practices. They were performed per cohort‐genotyping platform pair. We filtered out related individuals (with second‐degree or closer relatives) using KING software in both biobanks [24]. Further details are described in Method S4 [24, 25, 26, 27, 28, 29, 30].

#### 2.5.1 UK Biobank

UKBB samples (version 3; March 2018) were genotyped for > 800 000 single‐nucleotide polymorphisms (SNPs) using either an Affymetrix UK BiLEVE Axiom array or an Affymetrix UKBB Axiom array. After QC and imputation, 130 950 European (White‐British) male individuals were determined to be eligible for validation analyses.

#### 2.5.2 Penn Medicine Biobank

The PMBB consists of 43 623 samples that have been genotyped with the GSA genotyping array. Among them, 21 935 male samples were eligible for our study. After QC and imputation, a total of 14 279 individuals of European (non‐Hispanic White) ancestry and 3691 individuals of African American (non‐Hispanic Black) ancestry were determined to be eligible for replication analyses.

### 2.6 Polygenic Risk Score

We generated PRS using a previously published model developed from a large‐scale PrCa genome‐wide association study (GWAS) of summary statistics (107 247 PrCa cases and 127 006 controls) conducted by Conti et al. [31] The PRS model was composed of 269 independent PrCa risk variants and obtained from the PGS Catalogue (ID: PGS000662) [32]. Individual PRSs were computed from beta coefficients as a weighted sum of risk alleles by applying PLINK version 2.0 [33].

### 2.7 Statistical Analysis

Demographic and clinical characteristics are presented as mean ± standard deviation (SD) or as number (percentage). Continuous variables were compared by Student's *t*‐test or the Mann–Whitney U test as appropriate. Categorical variables were compared by the chi‐square test or Fisher's exact test as appropriate.

We used multivariate Cox proportional hazards regression models to evaluate the association of PrCA PRS with PrCa occurrence in groups according to each T2DM status. In the primary analysis, we calculated HRs and 95% confidence intervals (CIs) after adjusting for age, sex, the first 10 principal components (PCs) of ancestry, and genotyping array type. To account for population stratification, analyses were adjusted for the first 10 ancestry PCs derived from genome‐wide genotype data (Method S4). To address potential competing risk bias, we conducted two‐step validation analyses. Step 1 quantified T2DM‐stratified mortality using contingency tables with relative risks and chi‐squared tests for (a) all‐cause mortality (*n* = 8053 events) and (b) cardiovascular mortality (*n* = 1749 events). Step 2 applied Fine‐Gray subdistribution hazard models treating these mortality outcomes as competing events against PrCa incidence, with T2DM as the primary exposure [34].

HRs of the PRS were analysed both as a continuous variable (per one‐SD increase) and as a categorical variable with the following risk groups: low (< 20%), intermediate (20%–80%), high (80%–99%), and very high (> 99%). Sensitivity analyses addressed time‐varying T2DM bias by excluding incident T2DM cases identified post‐baseline, in addition to onset age, baseline age, and T2DM status stratified analyses. We further tested T2DM definition robustness across four criteria, ranging from Definition 1 (broad union of diagnosis, medication, glucose, and HbA1c) to 4 (all criteria required), and stratified T2DM by baseline HbA1c categories (< 48, 48–52, 53–63, and ≥ 64 mmol/mol). To assess potential non‐linear associations between sex hormone levels, IGF‐1 and incident PrCa risk, we applied restricted cubic spline functions within Cox regression models. Mediation analysis was conducted using the R ‘mediation’ package (v4.5.0), with prevalent T2DM as exposure, IGF‐1 as mediator, and incident PrCa as outcome. Nonparametric bootstrap (1000 simulations) estimated indirect effects, with multiple‐testing correction applied via Benjamini‐Hochberg procedure [35].

All statistical tests were two‐sided, and *p* < 0.05 was considered statistically significant. All statistical analyses were conducted using the R Statistical Software (version 4.5.0; R Foundation for Statistical Computing, Vienna, Austria) and PLINK version 2.0 [33].

## 3 Results

### 3.1 Study Population

A total of 130 950 men of European descent from the UKBB were included after excluding those with a history of any prevalent cancer or a lack of information concerning DM. The mean age of participants was 56.3 years (SD: 8.1 years). Results of comparing characteristics of participants in each group are presented in Table 1 and Table S1. In the replication set, we included 17 970 men from the PMBB cohort, comprising 14 279 of European descent and 3691 of African American descent (Table S1). The mean age of participants was 58.3 years (SD: 14.4 years). Detailed demographic information is shown in Figure S1.

**TABLE 1:** Demographic characteristics in the UK Biobank.

	All	Control	Incident PrCa	*p* ^a^
	(*N* = 130 950)	(*N* = 122 836)	(*N* = 8114)	
Age (baseline)	56.3 ± 8.1	56.0 ± 8.1	61.7 ± 5.6	< 0.001
Education year	14.3 ± 5.2	14.3 ± 5.1	14.1 ± 5.3	0.019
Townsend deprivation index	−1.5 ± 3.0	−1.5 ± 3.0	−1.9 ± 2.7	< 0.001
Number in household	2.5 ± 1.3	2.5 ± 1.3	2.2 ± 1.0	< 0.001
Average total household income before tax				< 0.001
less than £18 000	22 015 (18.6%)	20 358 (18.3%)	1657 (22.9%)	
£18 000 to £29 999	28 294 (23.9%)	26 282 (23.7%)	2012 (27.9%)	
£30 000 to £51 999	32 820 (27.8%)	30 931 (27.9%)	1889 (26.1%)	
£52 000 to £100 000	27 741 (23.5%)	26 439 (23.8%)	1302 (18.0%)	
greater than £100 000	7378 (6.2%)	7014 (6.3%)	364 (5.0%)	
SHBG	39.5 ± 16.5	39.4 ± 16.5	41.6 ± 16.2	< 0.001
Testosterone	12.0 ± 3.7	12.0 ± 3.7	11.9 ± 3.5	< 0.001
Free Testosterone	0.20 ± 0.1	0.20 ± 0.1	0.19 ± 0.1	< 0.001
Bioavailable Testosterone	5.3 ± 1.6	5.3 ± 1.6	5.0 ± 1.4	< 0.001
IGF‐1	22.0 ± 5.4	22.0 ± 5.4	21.7 ± 5.3	< 0.001

Abbreviations: IGF‐1, Insulin‐like growth factor 1; PrCa, prostate cancer; SHBG, sex hormone‐binding globulin.

^a^ *p*‐value indicates the significance of the difference between the control group and individuals diagnosed with incident prostate cancer.

### 3.2 Association Between Type 2 Diabetes Mellitus and Incident Prostate Cancer Risk

We confirmed that prevalent T2DM was significantly associated with a decreased risk of incident PrCa (HR, 0.75; 95% CI: 0.68–0.82; *p* < 0.001) in the UKBB (Table 2). This association remained significant regardless of the subgroup according to the onset age of PrCa. The HR was 0.57 (95% CI: 0.36–0.89) for early‐onset (≤ 60 years) and 0.76 (95% CI: 0.69–0.83) for late‐onset (> 60 years) of PrCa. Furthermore, a history of T2DM consistently has protective effects for PrCa across elderly or younger ones according to age. The protective effect due to T2DM was slightly stronger for those with younger age than for those with older age (HR: 0.70 [age ≤ 60] vs. 0.76 [age > 60]). The protective association robust to time‐varying exposure excluding incident T2DM maintained protective effect (HR 0.75, 95% CI 0.69–0.83, *p* = 4.39E‐09; Table S1).

**TABLE 2:** Hazard ratios for incident prostate cancer according to type 2 diabetes status (primary exposure) by age at cancer onset and baseline age in the UK Biobank and Penn Medicine Biobank.

	UK Biobank (UK)				Penn Medicine Biobank (USA)			
	Men (*n* = 130 950)				Men (*n* = 17 970)			
	No. of case/Total No.	HR	95% CI	*p*	No. of case/Total No.	HR	95% CI	*p*
All								
T2DM Absent	7655/123684	1 (reference)			878/14194	1 (reference)		
T2DM Present	459/7266	0.75	0.68–0.82	1.44E‐09	163/2735	0.67	0.57–0.80	5.92E‐06
Incident PrCa (onset ≤ 60)								
T2DM Absent	848/116877	1 (reference)			209/7420	1 (reference)		
T2DM Present	20/6827	0.57	0.36–0.89	1.31E‐02	30/1013	0.66	0.44–0.97	3.42E‐02
Incident PrCa (onset > 60)								
T2DM Absent	6807/122836	1 (reference)			669/6774	1 (reference)		
T2DM Present	439/7246	0.76	0.69–0.83	1.17E‐08	133/1722	0.67	0.55–0.81	2.65E‐05
Age ≤ 60								
T2DM Absent	2616/78823	1 (reference)			172/14194	1 (reference)		
T2DM Present	98/3192	0.70	0.57–0.86	5.21E‐04	22/2735	0.60	0.38–0.94	2.50E‐02
Age > 60								
T2DM Absent	5039/44861	1 (reference)			706/14194	1 (reference)		
T2DM Present	361/4074	0.76	0.69–0.85	7.79E‐07	141/2735	0.69	0.58–0.83	1.00E‐04

*Note:* Cox proportional hazard models were adjusted by age, genetic PC 1 to 10, and ethnicity (Penn Medicine Biobank).

Abbreviations: CI, confidence interval; HR, hazard ratio; PC, principal component; PrCa, prostate cancer; T2DM, type 2 diabetes mellitus.

T2DM patients exhibited significantly higher mortality (all‐cause: relative risk [RR] 2.87, χ^2^ *p* < 0.001; cardiovascular: RR 3.67, χ^2^ *p* < 0.001), confirming elevated competing risk potential (Table S1). However, Fine‐Gray models yielded PrCa HRs (all‐cause competing, HR: 0.74, 95% CI: 0.68–0.82; cardiovascular competing, HR: 0.74, 95% CI: 0.68–0.82) statistically indistinguishable from the standard Cox model (HR: 0.75, 95% CI: 0.68–0.82), with fully overlapping CIs (Table S1). Notably, this inverse association persisted across alternative T2DM definitions (HR: 0.74–0.85, all *p* < 0.002; Table S1). In the HbA1c‐stratified analyses, well‐controlled T2DM (HbA1c 6.5%–6.9%) showed attenuated protection (*p* = 0.29), whereas moderate/poor glycaemic control maintained significant protection (*p* < 0.001; Table S1).

For the replication set, the protective effect was replicated in an independent cohort, the PMBB. Males with a history of T2DM exhibited a 33% lower risk of PrCa (HR: 0.67; 95% CI: 0.57–0.80; *p* < 0.001) than participants without such a T2DM history. This trend was consistent regardless of PrCa onset age or baseline age (Table 2).

### 3.3 Analysis of Sex Hormones Related to the Incidence of Prostate Cancer

Based on existing hypotheses, we investigated relationships between sex hormone biomarkers (SHBG [sex hormone‐binding globulin], TT, bioT [bioavailable testosterone], and freeT [free testosterone]), the prevalence of T2DM, and the incidence of PrCa within the UKBB. At baseline, we confirmed that the group with prevalent T2DM exhibited significantly lower levels of all sex hormone biomarkers (SHBG, TT, bioT, and freeT) than the control group (all *p* < 0.001) (Table S1).

A lower level of SHBG was associated with an increased risk of PrCa (HR perSD increase: 0.93; 95% CI: 0.91–0.96; *p* < 0.001) among all male participants (Table S1). Conversely, bioT (HR perSD increase: 1.05; 95% CI: 1.03–1.08; *p* < 0.001) and freeT (HR perSD increase: 1.05; 95% CI: 1.02–1.07; *p* < 0.001) demonstrated a positive correlation with an increased risk of PrCa. TT showed no significant association with the risk of incident PrCa. We then conducted a mutually adjusted Cox proportional hazard regression analysis. As a result, the only association with SHBG for incident PrCa remained significant (Table S1). However, no sex hormone biomarkers associated significantly with incident PrCa were identified in participants with a history of T2DM (Figure S1).

### 3.4 Analysis of IGF‐1 Related to the Incidence of Prostate Cancer

At baseline, IGF‐1 levels were significantly lower in the group with prevalent T2DM than in the control group (*p* < 0.001) as shown in Table S1.

**FIGURE 1:**
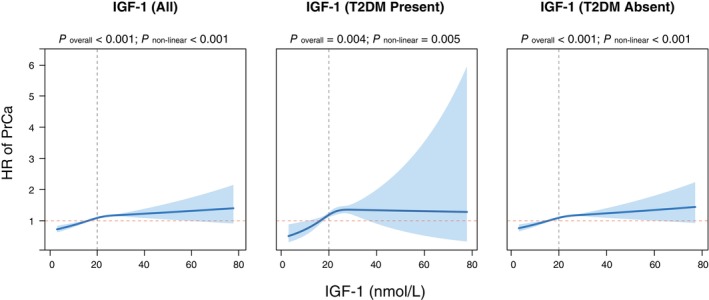
Restricted cubic spline analysis of the association between IGF‐1 and incident prostate cancer risk. T2DM, Type 2 diabetes mellitus; IGF‐1, Insulin‐like Growth Factor‐1; PrCa, prostate cancer.

In the entire male population, a higher level of IGF‐1 was associated with an increased risk of PrCa (HR perSD increase: 1.07; 95% CI: 1.05–1.09; *p* < 0.001) (Table S1). In subgroup analyses according to the presence of diabetes status, the significant association of identified IGF‐1 levels with PrCa risk was maintained in participants without a history of DM. A positive association between IGF‐1 levels and incident PrCa was also identified in participants with a history of DM. Hazard ratios for IGF‐1 levels according to baseline age are presented in Figure 1 and Table S1. Mediation analysis confirmed IGF‐1 mediates 5.1% (95% CI 3.5%–7.7%, *p* < 0.001) of T2DM's inverse association with PrCa risk (Table S1).

### 3.5 Association of PRS With Incident Prostate Cancer and Validation

The PRS model was composed of 269 independent PrCa risk variants, of which matched 243 variants were eligible in the PRS calculation for our study [22].

To validate the performance of the generated PrCa PRS, we utilized genotyped data from male participants within the UKBB (validation set) and PMBB (replication set) cohorts. PrCa PRS demonstrated a significant association (HR perSD increase: 1.77; 95% CI: 1.73–1.81; *p* < 0.001) with incident PrCa in the UKBB (Table S1).

These associations were replicated in the PMBB cohort. PRS for PrCa showed a significant association with incident PrCa (HR perSD increase: 1.62; 95% CI: 1.52–1.72; *p* < 0.001). This significance was maintained in population‐stratified analyses for European (HR perSD increase: 1.66; 95% CI: 1.55–1.79; *p* < 0.001) and African American (HR perSD increase: 1.47; 95% CI: 1.30–1.65; *p* < 0.001) populations (Table S1).

### 3.6 Impact of PRS on Prostate Cancer Risk Across Diabetes Status

**FIGURE 2:**
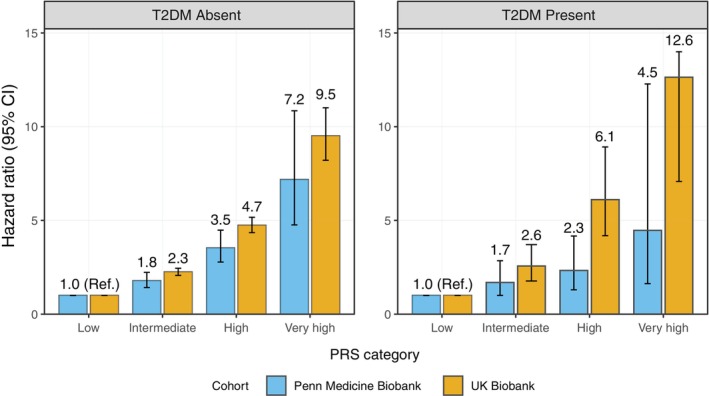
Hazard ratios for prostate cancer by polygenic risk score categories stratified by type 2 diabetes status. Bar heights represent cohort‐averaged HRs (UK Biobank [orange] and Penn Medicine Biobank [blue]) with 95% confidence intervals (error bars). PRS, polygenic risk score; HR, hazard ratio; T2DM, type 2 diabetes mellitus; CI, confidence interval.

In the UKBB, PrCa PRS showed a significant association with incident PrCa in male participants with a history of T2DM. The very high genetic risk group had a 12.64‐fold higher risk compared to the low genetic risk group. Among participants without a T2DM history, the PRS was a significant risk factor for incident PrCa, with the very high genetic risk group having a 9.51‐fold increased risk (95% CI: 8.21–11.01) compared to the low genetic risk group (Figure 2 and Table S1). Additionally, natural cubic spline analysis confirmed a monotonic dose–response relationship across the full continuum of PRS percentiles (*p* < 0.001; Figure S1). PRS quantile‐specific effect sizes are shown in Table S1.

For the replication set, PrCa PRS was also a significant risk factor for incident PrCa regardless of T2DM history in the PMBB. Among participants with a T2DM history, the very high genetic risk group had an HR of 4.47 (95% CI: 1.63–12.28) compared to the low genetic risk group. For participants without a T2DM history, the very high genetic risk group had a 7.18‐fold higher risk (95% CI: 4.76–10.85) compared to the low genetic risk group (Table S1).

In both cohorts, the PrCa PRS demonstrated a significant positive association with the incidence of PrCa, regardless of the presence of T2DM history.

### 3.7 Joint Associations of Prostate Cancer PRS and Diabetes Status

Within the UKBB, participants without a history of T2DM but with a very high genetic risk exhibited a 15.09‐fold higher risk of PrCa (95% CI: 10.44–21.80) compared to the reference group (low genetic risk group with a history of T2DM) (Figure S1). This trend was replicated in the PMBB, where participants without T2DM but with a very high genetic risk had a 9.24‐fold increase in the risk of PrCa (95% CI: 5.10–16.74) compared to the reference group. The multiplicative interaction term between DM history and PRS groups was not significant (*p* > 0.05) in either cohort.

### 3.8 Prediction Accuracy and Clinical Utility of Prostate Cancer PRS

The integration of PrCa PRS into conventional risk models significantly enhanced PrCa prediction accuracy across all additive models (Models 1–4). Cox proportional hazards models demonstrated that PRS addition improved discriminative performance, with C‐index values increasing from 0.709–0.716 to 0.758–0.763 (Table 3). Continuous net reclassification index (NRI) analyses indicated an improvement in risk stratification accuracy of 0.444–0.449 (all *p* < 0.001), independent of adjustments for sociodemographic factors, medical conditions or metabolic comorbidities.

**TABLE 3:** Model performance statistics for conventional risk factors and genetic risk for prostate cancer.

	(1) Reference model	(2) PRS‐additive model	(1) vs. (2)		
	C‐index(95% CI)	C‐index(95% CI)	Difference in C‐index (95% CI)	Continuous NRI (95% CI)	*p*
Model 1	0.709 (0.704–0.715)	0.758 (0.753–0.764)	0.049 (0.049–0.049)	0.449 (0.423–0.474)	< 0.001
Model 2	0.713 (0.707–0.719)	0.761 (0.755–0.766)	0.048 (0.048–0.047)	0.448 (0.421–0.475)	< 0.001
Model 3	0.715 (0.709–0.721)	0.762 (0.756–0.768)	0.047 (0.047–0.047)	0.445 (0.417–0.473)	< 0.001
Model 4	0.716 (0.709–0.722)	0.763 (0.757, 0.768)	0.047 (0.048–0.046)	0.444 (0.416–0.471)	< 0.001

*Note:* Model 1 included type 2 diabetes mellitus, age, genotype array, genetic PC 1 to 10, and IGF‐1. Model 2: Model 1 + education years + household income + Townsend deprivation index + number in household. Model 3: Model 2 + medical condition (body mass index + height + weight + waist circumference + systolic blood pressure + diastolic blood pressure + Total cholesterol + HDL cholesterol + LDL cholesterol + Triglycerides + Creatinine + eGFR + Fasting blood glucose + HbA1c). Model 4: Model 3 + prevalent metabolic disease (hypercholesterolaemia, hypertension, heart failure, chronic kidney disease, any stroke, diabetic hypoglycemia, and coronary artery disease).

Abbreviations: CI, confidence interval; HR, hazard ratio; IGF‐1, Insulin‐like growth factor 1; NRI, net reclassification index; PC, principal component; PrCa, prostate cancer; PRS, polygenic risk score; SD, standard deviation.

### 3.9 Clinical Scenarios for PRS‐Stratified Risk Assessment

PRS effectively stratifies prostate cancer risk across T2DM strata, enabling tailored screening strategies (Table S1). In T2DM patients, the top PRS percentile (≥ 99th) confers a 12.32‐fold increased risk despite T2DM's protective HR of 0.75, while non‐T2DM men at this genetic threshold face a 15.09‐fold risk (Figure S1). T2DM Present/low PRS men (≤ 20th percentile) exhibit minimal risk (2.19%), ideal for de‐intensified PSA screening (e.g., biennial intervals), whereas T2DM Absent/very high PRS men (20.08%) warrant intensified monitoring (e.g., annual PSA + magnetic resonance imaging).

## 4 Discussion

This large‐scale, multi‐cohort study leveraging the UKBB and PMBB demonstrates that T2DM is significantly associated with a reduced risk of PrCa. Across two independent cohorts, the PRS showed a consistent and strong association with PrCa risk in both diabetic and non‐diabetic individuals. Importantly, joint modelling confirmed that non‐diabetic individuals with very high genetic risk had the greatest incidence of PrCa and incorporation of the PRS into conventional risk models led to significant and consistent improvements in discrimination and reclassification.

PRS has been shown to enhance PrCa risk stratification, including for aggressive and early‐onset disease [36, 37, 38]. However, few studies have specifically examined PRS performance in individuals with T2DM. Prior case–control data suggested that lower PRS was associated with reduced PrCa risk among patients with T2DM [39] and UKBB‐based analyses across multiple cancer types have demonstrated substantial improvements in prediction accuracy for PrCa following PRS integration [40]. Our findings extend these observations by demonstrating robust PRS effects across diabetic and non‐diabetic populations in two independent biobanks and by quantifying improvements in C‐index and continuous NRI under progressively adjusted models that account for socioeconomic factors, metabolic comorbidities and T2DM status. Unlike prior single‐cohort analyses, our study provides replication and evaluates joint genetic–metabolic effects, strengthening the generalizability and robustness of the results.

Our two‐cohort design enabled consistency assessment across ancestries. The UKBB primary analyses used a large White British cohort (*n* = 130 950), while PMBB replication involved smaller European (*n* = 14 279) and African American (*n* = 3691) cohorts with wider CIs and attenuated effect sizes. Despite cohort differences (prospective vs. hospital‐based, size, phenotyping), directional consistency persisted, reflecting European GWAS‐trained PRS limitations in diverse populations. These findings support PRS utility across T2DM status, warranting ancestrally diverse validation.

Regarding hormonal mechanisms, previous genetic and epidemiologic studies have suggested that higher circulating testosterone levels may reduce T2DM risk while increasing PrCa risk [41]. Consistent with earlier reports, we observed lower baseline total, bioavailable, and free testosterone levels among individuals with T2DM. However, none of these sex hormone biomarkers was significantly associated with PrCa incidence within the diabetic population. These findings suggest that circulating sex hormones may not play a major mediating role in the inverse association between T2DM and PrCa risk, although limited statistical power and reliance on single baseline measurements should be considered. Despite objectively higher competing mortality in T2DM patients, Fine‐Gray sensitivity analyses confirmed that competing risk bias does not materially affect the protective T2DM‐PrCa association.

Circulating IGF‐1 has been consistently implicated in prostate carcinogenesis, with Mendelian randomization and large prospective studies reporting positive associations with overall and aggressive PrCa risk [42, 43]. In line with these data, we observed a robust positive association between IGF‐1 and incident PrCa. Notably, both linear and non‐linear dose–response patterns were identified in individuals with and without T2DM, suggesting a potentially complex biological gradient. Because baseline IGF‐1 levels were significantly lower among patients with T2DM, our mediation analysis indicates that IGF‐1 may partially mediate the inverse association between T2DM and PrCa. However, the mediation proportion was modest (5.1%), indicating that additional biological pathways likely contribute and warrant further longitudinal and mechanistic investigation.

This study has several limitations that should be considered in further studies. First, detection bias may occur in T2DM patients due to differences in the frequency of tests such as PSA tests, imaging studies and prostate biopsies, leading to a reduction in PrCa risk. The UKBB and PMBB data used in this study did not allow confirmation of differences in PSA screening frequency or the proportion undergoing biopsy after PSA elevation. Second, as the UKBB primarily consists of individuals of European ancestry, the generalizability of our findings to non‐European populations may be limited. Although our replication in the PMBB, which includes European American and African American participants, showed directional consistency, effect sizes and confidence intervals varied. These differences likely reflect the distinct cohort designs and the influence of polygenic scores derived predominantly from European‐based GWAS. Further validation in larger and ancestrally diverse populations will be essential to confirm the robustness of our findings. Third, PrCa outcomes in this study were defined using ICD codes when analysing biobank data. Therefore, data such as tumour stage and grade, Gleason score and metastatic status could not be confirmed. Consequently, we were unable to assess the aggressiveness of prostate cancer, which is clinically relevant when evaluating risk stratification strategies. Lastly, PRS represent upstream germline susceptibility rather than direct biological effectors. A high PRS does not guarantee prostate cancer development without tissue‐specific gene expression, epigenetic regulation and environmental interactions. Although our study demonstrates robust statistical associations between PRS and prostate cancer risk, functional validation linking PRS loci to prostate‐specific processes is lacking. Future studies with tissue‐specific expression quantitative trait loci and experimental validation are needed.

In conclusion, this large‐scale, multi‐cohort study across UKBB and PMBB demonstrates that PrCa PRS effectively stratifies PrCa risk independently of T2DM status, leading to significant improvements in prediction accuracy. These findings suggest that integrating PRS with diabetes status may enhance the precision of risk assessment, thereby warranting further studies to evaluate feasibility, clinical utility and cost‐effectiveness.

## Author Contributions

Guk Jin Lee, Sang‐Hyuk Jung, Jae‐Seung Yun and Dokyoon Kim conceived and designed the study. Guk Jin Lee and Sang‐Hyuk Jung performed statistical analyses and wrote the manuscript. Sang‐Hyuk Jung performed genetic data analysis. Guk Jin Lee and Jae‐Seung Yun curated the data. Sang‐Hyuk Jung, Jonghyun Lee and Ki Won Moon conducted data pre‐processing. Guk Jin Lee and Sang‐Hyuk Jung interpreted the data. Jonghyun Lee, Ki Won Moon and Jae‐Seung Yun read and critically revised the manuscript for intellectual content; all authors have read and approved the final manuscript. Jae‐Seung Yun and Dokyoon Kim supervised the project.

## Funding

This work was supported by the National Institute of Health (NIH) R01 GM138597 and R01 HL169458; the Korea Health Technology R&D Project through the Korea Health Industry Development Institute funded by the Ministry of Health and Welfare, Republic of Korea (No. RS‐2025‐24 535 069); the Regional Innovation System and Education (RISE) program through the Gangwon RISE Center, funded by the Ministry of Education (MOE) and the Gangwon State (G.S.), Republic of Korea (No. 2026‐RISE‐10‐002).

## Ethics Statement

This study complies with all relevant ethical regulations for research involving human participants. It was conducted in accordance with the criteria set by the Declaration of Helsinki. The UK Biobank (UKBB) was approved by the National Research Ethics Committee (June 17, 2011 [RES reference 11/NW/0382]; extended on May 10, 2016 [RES reference 16/NW/0274]). The present research using the UKBB Resource was approved under Application Number 90981. The collection, storage and analysis of biospecimens, genetic data, and data derived from electronic health records as part of the Penn Medicine Biobank (PMBB) were approved under University of Pennsylvania IRB protocol #813913. Participants from the UKBB and PMBB provided written informed consent allowing use of their samples and data for medical research purposes. This study followed the reporting requirements of the Strengthening the Reporting of Observational Studies in Epidemiology (STROBE) Statement.

## Consent

The authors have nothing to report.

## Conflicts of Interest

The authors declare no conflicts of interest.

## Supporting information


**Data S1:** dom70690‐sup‐0001‐Supinfo.docx.
**Supplementary Method 1**. Penn Medicine Biobank banner author list and contribution statements.
**Supplementary Method 2**. Detailed information on laboratory assays and data collection.
**Supplementary Method 3**. Missing data counts for biomarkers in the UK Biobank.
**Supplementary Method 4**. Detailed information on the genotype data quality control and imputation procedures.
**Supplementary Table 1**. Demographic characteristics according to diabetes status in the UK Biobank.
**Supplementary Table 2**. Demographic characteristics in the Penn Medicine Biobank.
**Supplementary Table 3**. Hazard ratios for incident prostate cancer by baseline type 2 diabetes status (primary exposure) excluding incident type 2 diabetes cases in the UK Biobank.
**Supplementary Table 4**. Type 2 diabetes mellitus‐stratified mortality contingency tables with relative risks and χ^2^ statistics.
**Supplementary Table 5**. Fine‐Gray competing risks analysis of type 2 diabetes and prostate cancer association accounting for mortality events.
**Supplementary Table 6**. Hazard ratios for incident prostate cancer according to alternative definitions of type 2 diabetes status in the UK Biobank.
**Supplementary Table 7**. Hazard ratios for incident prostate cancer by type 2 diabetes (primary exposure) according to HbA1c categories in the UK Biobank.
**Supplementary Table 8**. Hazard ratios for incident prostate cancer according to sex hormone levels stratified by type 2 diabetes status in the UK Biobank.
**Supplementary Table 9**. Hazard ratios for incident prostate cancer according to serum IGF‐1 levels (primary exposure) stratified by type 2 diabetes status in the UK Biobank.
**Supplementary Table 10**. Hazard ratios for incident prostate cancer according to serum IGF‐1 levels (primary exposure) stratified by baseline age in the UK Biobank.
**Supplementary Table 11**. Mediation analysis of the association between type 2 diabetes mellitus and incident prostate cancer risk mediated through baseline IGF‐1 levels.
**Supplementary Table 12**. Adjusted hazard ratios for incident prostate cancer according to prostate cancer polygenic risk score (primary exposure) in the UK Biobank.
**Supplementary Table 13**. Hazard ratios for incident prostate cancer according to prostate cancer polygenic risk score (primary exposure) stratified by ethnicity in the Penn Medicine Biobank.
**Supplementary Table 14**. Hazard ratios for incident prostate cancer according to prostate cancer polygenic risk score (primary exposure) stratified by type 2 diabetes status in the UK Biobank.
**Supplementary Table 15**. Dose–response associations of PRS quantiles with prostate cancer risk.
**Supplementary Table 16**. Clinical risk scenarios: polygenic risk score‐type 2 diabetes stratified 10‐year prostate cancer absolute risks and NCCN/EAU‐adapted screening recommendations.
**Supplementary Figure 1**. Demographic information for the UK Biobank and Penn Medicine Biobank.
**Supplementary Figure 2**. Restricted cubic spline analysis of the association between sex hormones and incident prostate cancer risk.
**Supplementary Figure 3**. Polygenic risk score distribution and incidence of prostate cancer risk by percentiles.
**Supplementary Figure 4**. Forest plots of risk of prostate cancer according to the genetic risk and diabetes mellitus status in the UK Biobank and Penn Medicine Biobank.

## Data Availability

The PrCa PRS model used in the current paper is available from the PGS Catalog (https://www.pgscatalog.org/score/PGS000662/). Biobank operational procedures are documented at https://github.com/normalhyuk/prca‐prs‐t2dm.
