# Supplementary material for: Polygenic Risk Score Predicts Prostate Cancer Risk Independent of Type 2 Diabetes
Source: Diabetes Obes Metab. 2026 Mar 26;28(6):4991–5000. doi: 10.1111/dom.70690 (PMC13146153; doi:10.1111/dom.70690)
Supplement: Supplementary file 1 — Data S1: dom70690‐sup‐0001‐Supinfo.docx. Supplementary Method 1. Penn Medicine Biobank banner author list and contribution statements. Supplementary Method 2. Detailed information on laboratory assays and data collection. Supplementary Method 3. Missing data counts for biomarkers in the UK Biobank. Supplementary Method 4. Detailed information on the genotype data quality control and imputation procedures. Supplementary Table 1. Demographic characteristics according to diabetes status in the UK Biobank. Supplementary Table 2. Demographic characteristics in the Penn Medicine Biobank. Supplementary Table 3. Hazard ratios for incident prostate cancer by baseline type 2 diabetes status (primary exposure) excluding incident type 2 diabetes cases in the UK Biobank. Supplementary Table 4. Type 2 diabetes mellitus‐stratified mortality contingency tables with relative risks and χ2 statistics. Supplementary Table 5. Fine‐Gray competing risks analysis of type 2 diabetes and prostate cancer association accounting for mortality events. Supplementary Table 6. Hazard ratios for incident prostate cancer according to alternative definitions of type 2 diabetes status in the UK Biobank. Supplementary Table 7. Hazard ratios for incident prostate cancer by type 2 diabetes (primary exposure) according to HbA1c categories in the UK Biobank. Supplementary Table 8. Hazard ratios for incident prostate cancer according to sex hormone levels stratified by type 2 diabetes status in the UK Biobank. Supplementary Table 9. Hazard ratios for incident prostate cancer according to serum IGF‐1 levels (primary exposure) stratified by type 2 diabetes status in the UK Biobank. Supplementary Table 10. Hazard ratios for incident prostate cancer according to serum IGF‐1 levels (primary exposure) stratified by baseline age in the UK Biobank. Supplementary Table 11. Mediation analysis of the association between type 2 diabetes mellitus and incident prostate cancer risk mediated through baseline IGF‐1 levels. S [file DOM-28-4991-s001.docx]

**Supplementary online material**

**Polygenic Risk Score Predicts Prostate Cancer Risk Independent of**

**Type 2 Diabetes**

**Supplementary Method 1.** Penn Medicine Biobank banner author list and contribution statements.

**Supplementary Method 2.** Detailed information on laboratory assays and data collection.

**Supplementary Method 3.** Missing data counts for biomarkers in the UK Biobank.

**Supplementary Method 4.** Detailed information on the genotype data quality control and imputation procedures.

**Supplementary Table 1.** Demographic characteristics according to diabetes status in the UK Biobank.

**Supplementary Table 2.** Demographic characteristics in the Penn Medicine Biobank.

**Supplementary Table 3.** Hazard ratios for incident prostate cancer by baseline type 2 diabetes status (primary exposure) excluding incident type 2 diabetes cases in the UK Biobank.

**Supplementary Table 4.** Type 2 diabetes mellitus-stratified mortality contingency tables with relative risks and χ² statistics.

**Supplementary Table 5.** Fine-Gray competing risks analysis of type 2 diabetes and prostate cancer association accounting for mortality events.

**Supplementary Table 6.** Hazard ratios for incident prostate cancer according to alternative definitions of type 2 diabetes status in the UK Biobank.

**Supplementary Table 7.** Hazard ratios for incident prostate cancer by type 2 diabetes (primary exposure) according to HbA1c categories in the UK Biobank.

**Supplementary Table 8.** Hazard ratios for incident prostate cancer according to sex hormone levels stratified by type 2 diabetes status in the UK Biobank.

**Supplementary Table 9.** Hazard ratios for incident prostate cancer according to serum IGF-1 levels (primary exposure) stratified by type 2 diabetes status in the UK Biobank.

**Supplementary Table 10.** Hazard ratios for incident prostate cancer according to serum IGF-1 levels (primary exposure) stratified by baseline age in the UK Biobank.

**Supplementary Table 11.** Mediation analysis of the association between type 2 diabetes mellitus and incident prostate cancer risk mediated through baseline IGF-1 levels.

**Supplementary Table 12.** Adjusted hazard ratios for incident prostate cancer according to prostate cancer polygenic risk score (primary exposure) in the UK Biobank.

**Supplementary Table 13.** Hazard ratios for incident prostate cancer according to prostate cancer polygenic risk score (primary exposure) stratified by ethnicity in the Penn Medicine Biobank.

**Supplementary Table 14.** Hazard ratios for incident prostate cancer according to prostate cancer polygenic risk score (primary exposure) stratified by type 2 diabetes status in the UK Biobank.

**Supplementary Table 15.** Dose-response associations of PRS quantiles with prostate cancer risk.

**Supplementary Table 16.** Clinical risk scenarios: polygenic risk score-type 2 diabetes stratified 10-year prostate cancer absolute risks and NCCN/EAU-adapted screening recommendations.

**Supplementary Figure 1.** Demographic information for the UK Biobank and Penn Medicine Biobank.

**Supplementary Figure 2.** Restricted cubic spline analysis of the association between sex hormones and incident prostate cancer risk.

**Supplementary Figure 3.** Polygenic risk score distribution and incidence of prostate cancer risk by percentiles.

**Supplementary Figure 4.** Forest plots of risk of prostate cancer according to the genetic risk and diabetes mellitus status in the UK Biobank and Penn Medicine Biobank.

**Supplementary References**

**Supplementary Method 1.** Penn Medicine Biobank banner author list and contribution statements.

**PMBB Leadership Team**

Daniel J. Rader, M.D., Marylyn D. Ritchie, Ph.D.

Contributions: All authors contributed to securing funding, study design, and oversight. All authors reviewed the final version of the manuscript.

**Patient Recruitment and Regulatory Oversight**

JoEllen Weaver, Nawar Naseer, Ph.D., M.P.H., Giorgio Sirugo, M.D., Ph.D., Afiya Poindexter, Yi-An Ko, Ph.D., Kyle P. Nerz.

Contributions: JW manages patient recruitment and regulatory oversight of the study. NN manages participant engagement and assists with regulatory oversight, and researcher access. GS assists with researcher access. AP, YK, and KPN recruit and enroll study participants.

**Lab Operations**

JoEllen Weaver, Meghan Livingstone, Fred Vadivieso, Stephanie DerOhannessian, Teo Tran, Julia Stephanowski, Salma Santos, Ned Haubein, Ph.D., Joseph Dunn.

Contributions: JW, ML, FV, and SD conduct oversight of lab operations. ML, FV, AK, SD, TT, JS, and SS perform sample processing. NH and JD are responsible for sample tracking and the laboratory information management system.

**Clinical Informatics**

Anurag Verma, Ph.D., Colleen Morse Kripke, M.S. DPT, MSA, Marjorie Risman, M.S., Renae Judy, B.S., Colin Wollack, M.S.

Contributions: All authors contributed to the development and validation of clinical phenotypes used to identify study subjects and (when applicable) controls.

**Genome Informatics**

Anurag Verma Ph.D., Shefali S. Verma, Ph.D., Scott Damrauer, M.D., Yuki Bradford, M.S., Scott Dudek, M.S., Theodore Drivas, M.D., Ph.D.

Contributions: AV, SSV, and SD are responsible for the analysis, design, and infrastructure needed to quality control genotype and exome data. YB performs the analysis. TD and AV provide variant and gene annotations and their functional interpretation of variants.

**Supplementary Method 2.** Detailed information on laboratory assays and data collection.

During the baseline assessment visit, fasting blood samples were collected into serum separator tubes according to standardized protocols, transported in temperature-controlled containers (4°C) [1] to a central processing laboratory, and stored at −80 °C. [2] Serum concentrations of testosterone, sex hormone-binding globulin (SHBG), and IGF-1 were measured for participants. Testosterone and SHBG levels were quantified using chemiluminescent immunoassays on a Beckman Coulter DXI 800 analyzer, while IGF-1 was measured using a DiaSorin Liaison XL platform. Free testosterone levels were estimated using a formula based on the law of mass action, incorporating measured values of total testosterone and SHBG. Detailed protocols for assay methodology and quality control procedures are available in the UKBB biomarker documentation (<https://biobank.ndph.ox.ac.uk/showcase/showcase/docs/serum_biochemistry.pdf>).

Potential confounders were addressed by collecting extensive covariate data at enrollment. Participants provided information on sociodemographic factors, medical history, and lifestyle behaviors through a self-administered touchscreen questionnaire and in-person baseline interviews. During the same visit, trained staff collected anthropometric measurements, including height, body weight, waist circumference, and hip circumference, using standardized protocols.

Blood samples were collected following established procedures [3] and processed centrally according to validated protocols [4]. HbA1c was measured using high-performance liquid chromatography on a Bio-Rad Variant II Turbo Analyzer. Levels serum glucose, lipids, and inflammatory markers were analyzed on a Beckman Coulter AU5800 platform using the following assays: hexokinase for glucose, CHO-POD for total cholesterol, GPO-POD for triglycerides, enzyme immunoinhibition for high-density lipoprotein (HDL) cholesterol, enzymatic selective protection for low-density lipoprotein (LDL) cholesterol, and immunoturbidimetric assays for lipoprotein (a) and high-sensitivity C-reactive protein.

Data of major chronic comorbidities were obtained from three sources: (1) participant self-reports via interviews or questionnaires at baseline; (2) diagnostic and procedural codes in linked electronic health records from hospital admissions; and (3) the first occurrence of diagnoses recorded in the UK Biobank health outcomes database, which integrates data from hospital inpatient records, cancer registries, primary care records, and death registries.

**Supplementary Method 3.** Missing data counts for biomarkers in the UK Biobank.

| **Biomarker** | **Missing (n)** | **Available (n [%])** |
| --- | --- | --- |
| (Total cohort reference) | - | 130,950 (100.0) |
| IGF-1 | 6,325 | 124,625 (95.2) |
| Total testosterone | 6,779 | 124,171 (94.8) |
| SHBG | 16,173 | 114,777 (87.6) |
| Bioavailable testosterone | 16,819 | 114,131 (87.2) |
| Free testosterone | 16,819 | 114,131 (87.2) |
| HbA1c | 3,446 | 127,504 (97.4) |
| Glucose | 15,435 | 115,515 (88.2) |

IGF-1, insulin-like growth factor 1; SHBG, sex hormone-binding globulin; HbA1c, hemoglobin A1C.

**Supplementary Method 4.** Detailed information on the genotype data quality control and imputation procedures.

***UK Biobank***

UK Biobank samples (version 3; March 2018) were genotyped for > 800,000 single-nucleotide polymorphisms (SNPs) using either the Affymetrix UK BiLEVE Axiom array or the Affymetrix UK Biobank Axiom array. Imputation was carried out centrally by UK Biobank researchers using the merged 1000 Genomes Project panel and UK 10K panel; SHAPEIT3 was used for phasing and IMPUTE2 was used for imputation (GRCh37/hg19) [5,6]. After imputation, variant-level quality control (QC) was performed by filtering SNPs on two criteria: (i) minor allele frequency < 1%, (ii) imputation quality score (INFO) < 0.3, and (iii) the Hardy–Weinberg equilibrium with a *P*-value of < 10^-6^. A total of 9,505,768 imputed autosomal SNPs passed the QC criteria. Sample-level QC was performed by excluding samples on the basis of (i) participants identified as not of ‘White-British’ ancestry according to either self-report or principal components (PC) analysis of genetic ancestry and (ii) mismatched sex and identified as not male. After exclusion, 130,950 White-British male participants were determined eligible for the genetic analyses.

***Penn Medicine Biobank***

Penn Medicine Biobank consists of 43,623 samples that have been genotyped by the GSA genotyping array. We performed genotype imputations for two Penn Medicine Biobank datasets using Eagle2 [7] and Minimac4 [8] softwares on the TOPMed Imputation Server [9]. Imputation was performed for all autosomes, with TOPMed version R2 on the GRCh38 reference panel [10]. After imputation, variant-level QC was performed by filtering SNPs on three criteria: 1) minor allele frequency <0.01, 2) marker call rate <0.05, and 3) INFO <0.2. Sample-level QC was performed by excluding samples on the basis of 1) mismatched sex or 2) having second-degree or closer relatives also in the Biobank. We inferred ancestry by projecting array genotype data onto PC axes defined by individuals from the 1000 Genome Project [11]. After exclusion, a total of 14,279 male participants considered European (EUR, non-Hispanic White) ancestry and 3,691 male participants considered African American (non-Hispanic Black) ancestry were determined eligible for the replication analyses.

**Supplementary Table 1.** Demographic characteristics according to diabetes status in the UK Biobank.

|  | **All** | **Control** | **Prevalent T2DM** | ***P*-value*** |
| --- | --- | --- | --- | --- |
|  | **(N=130,950)** | **(N=123,684)** | **(N=7,266)** |  |
| Age (baseline) | 56.3 ± 8.1 | 56.1 ± 8.1 | 60.1 ± 6.7 | <0.001 |
| Education year | 14.3 ± 5.2 | 14.3 ± 5.1 | 13.1± 5.2 | <0.001 |
| Townsend deprivation index | -1.5 ± 3.0 | -1.6 ± 3.0 | -0.9 ± 3.3 | <0.001 |
| Number in household | 2.5 ± 1.3 | 2.5 ± 1.3 | 2.2 ± 1.0 | <0.001 |
| Average total household income before tax |  |  |  | <0.001 |
| less than £18,000 | 22,015 (18.6%) | 19962 (17.8%) | 2053 (32.2%) |  |
| £18,000 to £29,999 | 28,294 (23.9%) | 26497 (23.7%) | 1797 (28.2%) |  |
| £30,000 to £51,999 | 32,820 (27.8%) | 31373 (28.0%) | 1447 (22.7%) |  |
| £52,000 to £100,000 | 27,741 (23.5%) | 26837 (24.0%) | 904 (14.2%) |  |
| greater than £100,000 | 7,378 (6.2%) | 7199 (6.4%) | 179 (2.8%) |  |
| SHBG | 39.5 ± 16.5 | 39.8 ± 16.4 | 34.8 ± 16.6 | <0.001 |
| Testosterone | 12.0 ± 3.7 | 12.1 ± 3.6 | 10.3 ± 3.5 | <0.001 |
| Free Testosterone | 0.20 ± 0.1 | 0.21 ± 0.1 | 0.19 ± 0.1 | <0.001 |
| Bioavailable Testosterone | 5.3 ± 1.6 | 5.3 ± 1.5 | 4.8 ± 1.5 | <0.001 |
| IGF-1 | 22.0 ± 5.4 | 22.1 ± 5.4 | 20.4 ± 6.4 | <0.001 |

**P*-value indicates the significance of the difference between the control group and individual diagnosed with prevalent type 2 diabetes mellitus.

T2DM, type 2 diabetes mellitus; SHBG, sex hormone-binding globulin; IGF-1, Insulin-like growth factor 1.

**Supplementary Table 2.** Demographic characteristics in the Penn Medicine Biobank.

|  | **All** | **Control** | **Incident PrCa** | ***P*-value*** |
| --- | --- | --- | --- | --- |
|  | **(N=17,970)** | **(N=16,929)** | **(N=1,041)** |  |
| Age (baseline) | 58.3 ± 14.4 | 57.8 ± 14.6 | 66.3 ± 8.1 | <0.001 |
| Ethnicity |  |  |  | <0.001 |
| European (non-Hispanic White) | 14,279 (79.5%) | 13,522 (79.9%) | 757 (72.7%) |  |
| African American (non-Hispanic Black) | 3,691 (20.5%) | 3,407 (20.1%) | 284 (27.3%) |  |

**P*-value indicates the significance of the difference between the control group and individual diagnosed with incident prostate cancer.

PrCa, prostate cancer.

**Supplementary Table 3**. Hazard ratios for incident prostate cancer by baseline type 2 diabetes status (primary exposure) excluding incident type 2 diabetes cases in the UK Biobank.

|  | **No. of case/**  **Total No.** | **HR** | **95% CI** | ***P*-value** |
| --- | --- | --- | --- | --- |
| **Main Analysis** | | | | |
| T2DM Absent | 7,655/123,684 | 1 (reference) |  |  |
| T2DM Present | 459/7,266 | 0.75 | 0.68-0.82 | 1.44E-09 |
| **Excluding incident T2DM (n=4,853)** | | | | |
| T2DM Absent | 7,236/118,831 | 1 (reference) |  |  |
| T2DM Present | 459/7,266 | 0.75 | 0.69-0.83 | 4.39E-09 |

Cox proportional hazard models were adjusted by age and genetic PC 1 to 10.

HR, hazard ratio; CI, confidence interval; T2DM, type 2 diabetes mellitus; PrCa, prostate cancer; PC, principal component.

**Supplementary Table 4.** Type 2 diabetes mellitus-stratified mortality contingency tables with relative risks and χ² statistics.

|  | **a. All-cause mortality** | | **b. Cardiovascular mortality** | |
| --- | --- | --- | --- | --- |
|  | **Death (0)** | **Death (1)** | **Death (0)** | **Death (1)** |
| **T2DM Absent** | 116,793 | 6,891 (5.6%) | 122,245 | 1,439 (1.2%) |
| **T2DM Present** | 6,104 | 1,162 (16.0%) | 6,956 | 310 (4.3%) |
| **Relative Risk** | Ref. | 2.87 | Ref. | 3.67 |
| **χ² *P*-value** |  | 2.19×10^-282^ |  | 1.50×10^-110^ |

T2DM, type 2 diabetes mellitus.

**Supplementary Table 5.** Fine-Gray competing risks analysis of type 2 diabetes and prostate cancer association accounting for mortality events.

| **Type 2 diabetes mellitus**  **(primary exposure)** | **Standard Cox (Baseline model)** | **All-cause mortality competing risk** | **Cardiovascular mortality competing risk** |
| --- | --- | --- | --- |
| **sHR (95% CI)** | 0.75 (0.68-0.82) | 0.74 (0.68-0.82) | 0.74 (0.68-0.82) |
| ***P*-value** | <0.001 | <0.001 | <0.001 |

All models adjusted for polygenic risk score for prostate cancer, age, genotyping array, and PCs 1-10.
sHR, subdistribution hazard ratio; CI, confidence interval; PC, principal component.

**Supplementary Table 6**. Hazard ratios for incident prostate cancer according to alternative definitions of type 2 diabetes status in the UK Biobank.

|  | **No. of case/**  **Total No.** | **HR** | **95% CI** | ***P*-value** |
| --- | --- | --- | --- | --- |
| **Definition 1 (Broad union)** | | | | |
| T2DM Absent | 7,655/123,684 | 1 (reference) |  |  |
| T2DM Present | 459/7,266 | **0.75** | 0.68-0.82 | 1.44E-09 |
| **Definition 2 (Diagnosis + medication)** | | | | |
| T2DM Absent | 7,655/116,029 | 1 (reference) |  |  |
| T2DM Present | 299/4,874 | **0.83** | 0.79-0.89 | 1.10E-09 |
| **Definition 3 (Diagnosis + medication + glucose)** | | | | |
| T2DM Absent | 7,624/115,233 | 1 (reference) |  |  |
| T2DM Present | 194/3,088 | **0.85** | 0.79-0.91 | 4.51E-06 |
| **Definition 4 (Strict criteria)** | | | | |
| T2DM Absent | 7,624/115,197 | 1 (reference) |  |  |
| T2DM Present | 28/644 | **0.74** | 0.62-0.90 | 1.85E-03 |

Definition 1 (Broad union): T2DM diagnosis codes OR glucose-lowering medication OR elevated random glucose (≥11.1 mmol/L) OR HbA1c ≥48 mmol/mol (6.5%)

Definition 2 (Diagnosis + medication): T2DM diagnosis codes AND glucose-lowering medication

Definition 3 (Diagnosis + medication + glucose): T2DM diagnosis codes AND glucose-lowering medication AND elevated random glucose (≥11.1 mmol/L)

Definition 4 (Strict criteria): T2DM diagnosis codes AND glucose-lowering medication AND elevated random glucose (≥11.1 mmol/L) AND HbA1c ≥48 mmol/mol (6.5%)

Cox proportional hazard models were adjusted by age and genetic PC 1 to 10.

HR, hazard ratio; CI, confidence interval; T2DM, type 2 diabetes mellitus; PC, principal component.

**Supplementary Table 7**. Hazard ratios for incident prostate cancer by type 2 diabetes (primary exposure) according to HbA1c categories in the UK Biobank.

| **HbA1c Category** | **No. cases/Total N** | **HR (95% CI)** | ***P*-value** |
| --- | --- | --- | --- |
| Normal (<48 mmol/mol, <6.5%) | 7,622/115,192 | 1 (reference) | - |
| Well-controlled (48-52 mmol/mol, 6.5-6.9%) | 98/1,224 | 0.89 (0.73-1.10) | 0.290 |
| Moderately controlled (53-63 mmol/mol, 7.0-7.9%) | 116/1,826 | 0.72 (0.59-0.88) | 1.08E-03 |
| Poorly controlled (≥64 mmol/mol, ≥8.0%) | 55/1,371 | 0.85 (0.79-0.91) | 2.42E-04 |

Cox proportional hazard models were adjusted by age and genetic PC 1 to 10.

HR, hazard ratio; CI, confidence interval; PC, principal component.

**Supplementary Table 8.** Hazard ratios for incident prostate cancer according to sex hormone levels stratified by type 2 diabetes status in the UK Biobank.

| **Primary exposure** | **Total No.** | **No. of Incident**  **PrCa cases (%)** | **HR**  **(perSD increase)** | **95% CI** | ***P*-value** | ***P*-value (mutual)*** |
| --- | --- | --- | --- | --- | --- | --- |
| **SHBG** |  |  |  |  |  |  |
| All | 114,777 | 7,125 (6.21%) | 0.93 | (0.91-0.96) | **1.09E-08** | **1.13E-02** |
| T2DM Present | 6,334 | 383 (6.05%) | 1.00 | (0.90-1.11) | 9.95E-01 | 9.39E-01 |
| T2DM Absent | 108,443 | 6,742 (6.22%) | 0.93 | (0.91-0.95) | **4.19E-09** | **5.93E-03** |
| **Testosterone** |  |  |  |  |  |  |
| All | 124,171 | 7,665 (6.17%) | 0.99 | (0.97-1.02) | 6.18E-01 | 3.12E-01 |
| T2DM Present | 6,874 | 415 (6.04%) | 1.06 | (0.97-1.17) | 2.04E-01 | 9.91E-01 |
| T2DM Absent | 117,297 | 7,250 (6.18%) | 0.99 | (0.97-1.01) | 4.17E-01 | 2.22E-01 |
| **Bioavailable Testosterone** |  |  |  |  |  |  |
| All | 114,131 | 7,075 (6.20%) | 1.05 | (1.03-1.08) | **4.98E-05** | 3.40E-01 |
| T2DM Present | 6,291 | 375 (5.96%) | 1.08 | (0.99-1.19) | 1.01E-01 | 3.46E-01 |
| T2DM Absent | 107,840 | 6,700 (6.21%) | 1.05 | (1.02-1.08) | **1.92E-04** | 4.89E-01 |
| **Free Testosterone** |  |  |  |  |  |  |
| All | 114,131 | 7,075 (6.20%) | 1.05 | (1.02-1.07) | **1.40E-04** | 2.96E-01 |
| T2DM Present | 6,291 | 375 (5.96%) | 1.07 | (0.98-1.17) | 1.40E-01 | 4.76E-01 |
| T2DM Absent | 107,840 | 6,700 (6.21%) | 1.05 | (1.02-1.07) | **4.42E-04** | 3.16E-01 |

Cox proportional hazard models were adjusted by type 2 diabetes mellitus, polygenic risk score for PrCa, age, genotype array, and genetic PC 1 to 10.

**P*-value estimates were derived from a mutual adjustments model (SHBG, testosterone, bioavailable testosterone, and free testosterone)

PrCa, prostate cancer; SHBG, Sex hormone binding globulin; T2DM, type 2 diabetes mellitus; HR, hazard ratio; CI, confidence interval; PC, principal component.

**Supplementary Table 9.** Hazard ratios for incident prostate cancer according to serum IGF-1 levels (primary exposure) stratified by type 2 diabetes status in the UK Biobank.

|  | **No. of Total** | **No. of Incident**  **PrCa cases (%)** | **HR**  **(perSD increase)** | **95% CI** | ***P*-value** |
| --- | --- | --- | --- | --- | --- |
| **IGF-1** |  |  |  |  |  |
| All | 124,625 | 7,703 (6.18%) | 1.07 | (1.05-1.09) | **4.79E-09** |
| T2DM Present | 6,906 | 423 (6.13%) | 1.15 | (1.05-1.26) | **3.40E-03** |
| T2DM Absent | 117,719 | 7,280 (6.18%) | 1.06 | (1.04-1.09) | **1.22E-07** |

Cox proportional hazard models were adjusted by type 2 diabetes mellitus, polygenic risk score for PrCa, age, genotype array, and genetic PC 1 to 10.

PrCa, prostate cancer; IGF-1, Insulin-like growth factor 1; T2DM, type 2 diabetes mellitus; HR, hazard ratio; CI, confidence interval; PC, principal component.

**Supplementary Table 10.** Hazard ratios for incident prostate cancer according to serum IGF-1 levels (primary exposure) stratified by baseline age in the UK Biobank.

|  | **No. of Total** | **No. of Incident**  **PrCa cases (%)** | **HR**  **(perSD increase)** | **95% CI** | ***P*-value** |
| --- | --- | --- | --- | --- | --- |
| **IGF-1** |  |  |  |  |  |
| **Baseline age (yr)** |  |  |  |  |  |
| **≤ 45** | 28,670 | 131 (0.5%) | 1.11 | 0.91-1.36 | 0.304 |
| **46-50** | 29,077 | 497 (1.7%) | 1.06 | 0.95-1.18 | 0.279 |
| **51-55** | 33,127 | 992 (3.0%) | 1.04 | 0.96-1.12 | 0.358 |
| **56-60** | 41,983 | 2,174 (5.2%) | **1.10** | **1.05-1.16** | **<.001** |
| **60-65** | 52,697 | 4,060 (7.7%) | **1.07** | **1.04-1.11** | **<.001** |
| **66 <** | 34,165 | 3,023 (8.8%) | **1.04** | **1.00-1.09** | **0.047** |

Cox proportional hazard models were adjusted by type 2 diabetes mellitus, polygenic risk score for PrCa, age, genotype array, and genetic PC 1 to 10.

PrCa, prostate cancer; IGF-1, Insulin-like growth factor 1; T2DM, type 2 diabetes mellitus; HR, hazard ratio; CI, confidence interval; PC, principal component.

**Supplementary Table 11.** Mediation analysis of the association between type 2 diabetes mellitus and incident prostate cancer risk mediated through baseline IGF-1 levels.

| **Effect Type** | **Estimate** | **95% CI** | ***P*-value** |
| --- | --- | --- | --- |
| ACME (indirect) | -0.001 | (-0.0014, -0.0008) | <0.001 |
| ADE (direct) | -0.020 | (-0.026, -0.014) | <0.001 |
| Total Effect | -0.021 | (-0.027, -0.015) | <0.001 |
| **Proportion Mediated** | **5.1%** | **(3.5%, 7.7%)** | **<0.001** |

Analysis performed using the R “mediation” package (Tingley et al., 2014) [12] with nonparametric bootstrap with 1,000 simulations. Models adjusted for age, PrCa PRS, genotyping array, and ancestry PCs 1-10.

ACME, average causal mediation effect; ADE, average direct effect; CI, confidence interval; PRS, polygenic risk score; PrCa, prostate cancer; PC, principal component.

**Supplementary Table 12.** Adjusted hazard ratios for incident prostate cancer according to genetic risk for prostate cancer in the UK Biobank.

|  |  |  | **Model 1** |  | **Model 2** |  | **Model 3** |  | **Model 4** |  |
| --- | --- | --- | --- | --- | --- | --- | --- | --- | --- | --- |
| **Genetic risk** | **Total no. of participants** | **No. of incident PrCa cases (%)** | **HR (95% CI)** | ***P*-value** | **HR (95% CI)** | ***P*-value** | **HR (95% CI)** | ***P*-value** | **HR (95% CI)** | ***P*-value** |
| **Low** | 26,200 | 670 (2.56%) | 1 (reference) | | 1 (reference) | | 1 (reference) | | 1 (reference) | |
| **Intermediate** | 78,570 | 4,420 (5.63%) | 2.26 (2.08-2.46) | <.001 | 2.27 (2.07-2.48) | <.001 | 2.26 (2.06-2.49) | <.001 | 2.26 (2.06-2.49) | <.001 |
| **High** | 24,871 | 2,758 (11.09%) | 4.78 (4.38-5.21) | <.001 | 4.83 (4.40-5.30) | <.001 | 4.83 (4.39-5.33) | <.001 | 4.83 (4.39-5.32) | <.001 |
| **Very high** | 1,309 | 266 (20.32%) | 9.58 (8.27-11.09) | <.001 | 9.54 (8.15-11.15) | <.001 | 9.42 (7.99-11.10) | <.001 | 9.41 (7.98-11.09) | <.001 |
| **perSD increase** |  |  | 1.77 (1.73-1.81) | <.001 | 1.77 (1.72-1.81) | <.001 | 1.76 (1.72-1.81) | <.001 | 1.76 (1.72-1.81) | <.001 |

Model 1 was adjusted by type 2 diabetes mellitus, age, genotype array, genetic PC 1 to 10, and IGF-1.

Model 2: Model 1 + education years + household income + Townsend deprivation index + number in household.

Model 3: Model 2 + medical condition (body mass index + height + weight + waist circumference + systolic blood pressure + diastolic blood pressure + Total cholesterol + HDL cholesterol + LDL cholesterol + Triglycerides + Creatinine + eGFR + Fasting blood glucose + HbA1c).

Model 4: Model 3 + prevalent metabolic disease (hypercholesterolaemia, hypertension, heart failure, chronic kidney disease, any stroke, diabetic hypoglycemia, and coronary artery disease)

PrCa, prostate cancer; SD, standard deviation; HR, hazard ratio; CI, confidence interval; PC, principal component; IGF-1, Insulin-like growth factor 1.

**Supplementary Table 13.** Hazard ratios for incident prostate cancer according to prostate cancer polygenic risk score (primary exposure) stratified by ethnicity in the Penn Medicine Biobank.

|  | **All male participants^1^** | | | | **European^2^**  (non-Hispanic White) | | **African American^2^**  (non-Hispanic Black) | |
| --- | --- | --- | --- | --- | --- | --- | --- | --- |
| **Genetic risk** | **Total no. of participants** | **No. of incident PrCa cases (%)** | **HR (95% CI)** | ***P*-value** | **HR (95% CI)** | ***P*-value** | **HR (95% CI)** | ***P*-value** |
| **Low** | 3,600 | 107 (3.0%) | 1 (reference) | | 1 (reference) | | 1 (reference) | |
| **Intermediate** | 10,790 | 568 (5.3%) | 1.77 (1.44-2.18) | <.001 | 1.74 (1.38-2.18) | <.001 | 1.81 (1.11-2.95) | .018 |
| **High** | 3,401 | 330 (9.7%) | 3.34 (2.68-4.17) | <.001 | 3.49 (2.73-4.47) | <.001 | 2.92 (1.76-4.83) | <.001 |
| **Very high** | 179 | 36 (20.11%) | 6.64 (4.53-9.72) | <.001 | 7.29 (4.56-11.66) | <.001 | 5.60 (2.78-11.31) | <.001 |
| **perSD increase** |  |  | 1.62 (1.52-1.72) | <.001 | 1.66 (1.55-1.79) | <.001 | 1.47 (1.30-1.65) | <.001 |

^1^Cox proportional hazard model was adjusted by type 2 diabetes mellitus, age, genetic PC 1 to 10, and ethnicity.

^2^Cox proportional hazard model was adjusted by type 2 diabetes mellitus, age, and genetic PC 1 to 10.

PrCa, prostate cancer; SD, standard deviation; HR, hazard ratio; CI, confidence interval; PC, principal component.

**Supplementary Table 14.** Hazard ratios for incident prostate cancer according to prostate cancer polygenic risk score (primary exposure) stratified by type 2 diabetes status in the UK Biobank.

|  | ***Hx of T2DM*** | | | | | ***No Hx of T2DM*** | | | | |
| --- | --- | --- | --- | --- | --- | --- | --- | --- | --- | --- |
| Genetic risk | Total no. of participants | No. of incident PrCa cases (%) | Incidence rate 1000 men-years  (95% CI) | HR (95% CI) | *P*-value | Total no. of participants | No. of incident PrCa cases (%) | Incidence rate 1000 men-years  (95% CI) | HR (95% CI) | *P*-value |
| ***UK Biobank*** | | | | | |  |  |  |  |  |
| Low | 1,461 | 32 (2.19%) | 1.81  (1.24-2.55) | 1 (reference) |  | 24,739 | 638(2.58%) | 2.13  (1.97-2.3) | 1 (reference) |  |
| Intermediate | 4,311 | 237 (5.50%) | 4.62  (4.05-5.25) | 2.57 (1.77-3.71) | <.001 | 74,259 | 4,183 (5.63%) | 4.72  (4.58-4.87) | 2.25 (2.07-2.45) | <.001 |
| High | 1,420 | 172 (12.11%) | 10.5  (8.99-12.19) | 6.11 (4.19-8.92) | <.001 | 23,451 | 2,586 (11.03%) | 9.54  (9.17-9.91) | 4.74 (4.35-5.17) | <.001 |
| Very high | 74 | 18 (24.32%) | 22.48  (13.32-35.52) | 12.64 (7.08-22.57) | <.001 | 1,235 | 248 (20.08%) | 18.24  (16.04-20.65) | 9.51 (8.21-11.01) | <.001 |
| perSD increase |  |  |  | 1.92 (1.75-2.11) | <.001 |  |  |  | 1.76 (1.72-1.80) | <.001 |
| ***Penn Medicine biobank*** | | | | | |  |  |  |  |  |
| Low | 564 | 17 (3.01%) | 8.67  (5.05-13.88) | 1 (reference) |  | 3,036 | 90 (3.05%) | 8.99  (7.23-11.06) | 1 (reference) |  |
| Intermediate | 1,780 | 100 (5.62%) | 16.5  (13.42-20.07) | 1.69 (1.00-2.85) | .050 | 9,010 | 468 (5.48%) | 15.93  (14.52-17.44) | 1.78 (1.42-2.23) | <.001 |
| High | 520 | 41 (7.88%) | 23.72  (17.02-32.18) | 2.33 (1.30-4.17) | .005 | 2,881 | 289 (11.15%) | 32.46  (28.82-36.42) | 3.53 (2.78-4.48) | <.001 |
| Very high | 34 | 5 (14.71%) | 44.41  (14.42-103.63) | 4.47 (1.63-12.28) | .004 | 145 | 31 (27.19%) | 70.64  (47.99-100.26) | 7.18 (4.76-10.85) | <.001 |
| perSD increase |  |  |  | 1.45 (1.23-1.70) | <.001 |  |  |  | 1.65 (1.54-1.77) | <.001 |

Cox proportional hazard models were adjusted by age, genotype array, and genetic PC 1 to 10.

T2DM, type 2 diabetes mellitus; PrCA, prostate cancer; SD, standard deviation; HR, hazard ratio; CI, confidence interval; PC, principal component.

**Supplementary Table 15.** Dose-response associations of PRS quantiles with prostate cancer risk.

| **PRS grouping** | **Comparison** | **HR (95% CI)** | ***P*-value** |
| --- | --- | --- | --- |
| **Tertiles (3)** | Q1 vs. Q2 | 1.88 (1.76-2.01) | <0.001 |
|  | Q1 vs. Q3 | 3.59 (3.38-3.82) | <0.001 |
| **Quartiles (4)** | Q1 vs. Q2 | 1.69 (1.55-1.83) | <0.001 |
|  | Q1 vs. Q3 | 2.45 (2.27-2.65) | <0.001 |
|  | Q1 vs. Q4 | 4.33 (4.03-4.66) | <0.001 |
| **Quintiles (5)** | Q1 vs. Q2 | 1.61 (1.46-1.78) | <0.001 |
|  | Q1 vs. Q3 | 2.22 (2.02-2.43) | <0.001 |
|  | Q1 vs. Q4 | 3.00 (2.75-3.27) | <0.001 |
|  | Q1 vs. Q5 | 5.02 (4.62-5.46) | <0.001 |

Q1 (lowest PRS quantile) as the reference group for all comparisons.

Cox proportional hazards models adjusted for type 2 diabetes mellitus, age, genotyping array, and ancestry PCs 1-10.

PRS, polygenic risk score; HR, hazard ratio; CI, confidence interval; PC, principal component.

**Supplementary Table 16.** Clinical risk scenarios: polygenic risk score-type 2 diabetes stratified 10-year prostate cancer absolute risks and NCCN/EAU-adapted screening recommendations.

| **Rank** | **Scenario** | **T2DM Status** | **PRS Percentile** | **10-Year Absolute Risk** | **Proportion Affected** | **HR vs. Low-Risk Reference (95% CI)** | **Clinical Recommendation**  **(NCCN/EAU-Adapted)** |
| --- | --- | --- | --- | --- | --- | --- | --- |
| 1 | Highest Risk | Absent | 99^th^  (Very High) | 20.08% | 1 in 5 | 15.09 (10.44-21.80) | Highest risk: PSA screening from age 45 years; annual PSA with mpMRI from age ≥50 years; immediate specialist referral. |
| 2 | High Risk | Present | 99^th^  (Very High) | 24.32% | 1 in 4 | 12.32 (6.91-21.94) | High risk despite T2DM: Initiate PSA screening at age 50 years; annual PSA with or without mpMRI; PRS-informed urology consultation. |
| 3 | Moderate Risk | Absent | 20-80^th^  (Intermediate) | 5.63% | 1 in 18 | 3.61 (2.55-5.11) | Standard screening: Shared decision-making for PSA screening ages 50-75 years; repeat every 2-4 years if PSA 1-3 ng/mL. |
| 4 | Moderate Risk | Present | 20-80^th^  (Intermediate) | 5.50% | 1 in 18 | 2.56 (1.77-3.70) | Average risk: Standard age-based screening; T2DM does not modify screening approach. |
| 5 | Low Risk | Absent | ≤20^th^  (Low) | 2.58% | 1 in 38 | 1.60 (1.12-2.28) | Low risk: Consider delaying screening; repeat PSA every 1-3 years or discontinue if PSA <1 ng/mL after age 75 years. |
| 6 | Lowest Risk | Present | ≤20^th^  (Low) | 2.19% | 1 in 45 | 1 (Reference) | Lowest risk: Screening deferral is clinically appropriate in the absence of other risk factors. |

PRS, polygenic risk score; HR, hazard ratio; CI, confidence interval; NCCN, National Comprehensive Cancer Network; EAU, European Association of Urology; mpMRI, Multi-parametric magnetic resonance imaging; PSA, prostate-specific antigen.

**Supplementary Figure 1.** Demographic information for the UK Biobank and Penn Medicine Biobank.


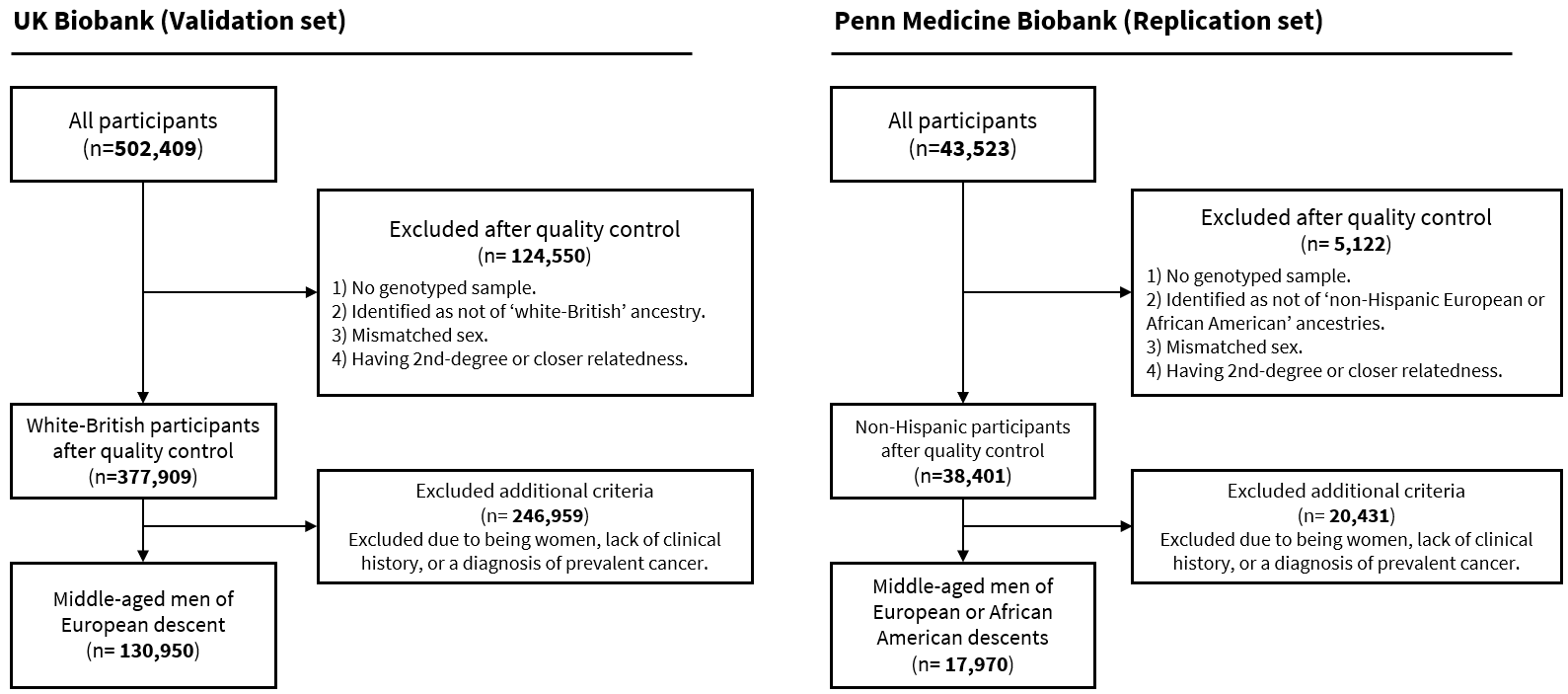


**Supplementary Figure 2.** Restricted cubic spline analysis of the association between sex hormones and incident prostate cancer risk.


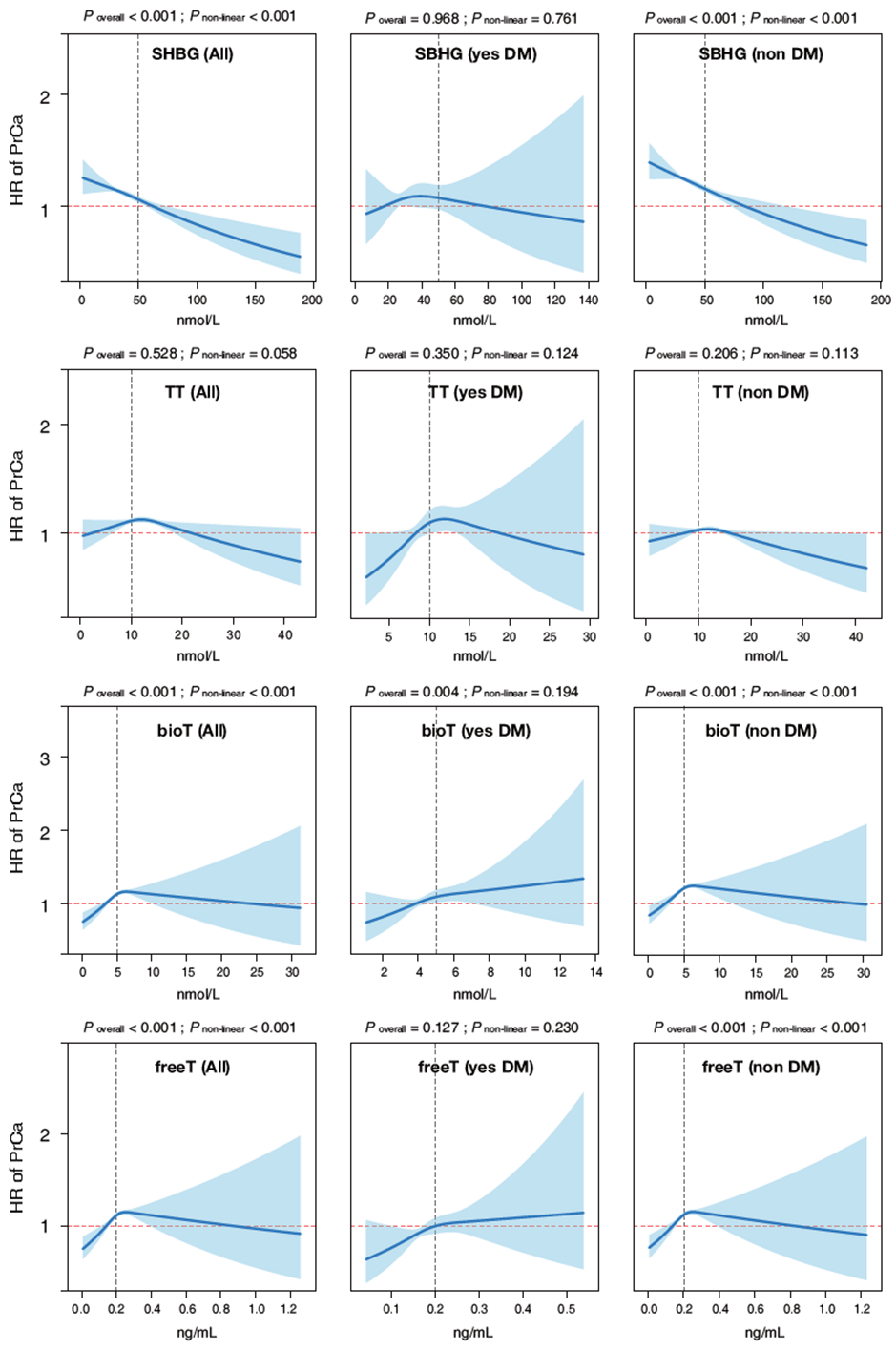


PrCa, Prostate Cancer; SHBG, Sex Hormone-Binding Globulin; TT, Testosterone; bioT, Bioavailable Testosterone; freeT, Free Testosterone; DM, Diabetes Mellitus.

**Supplementary Figure 3.** Polygenic risk score distribution and incidence of prostate cancer risk by percentiles.


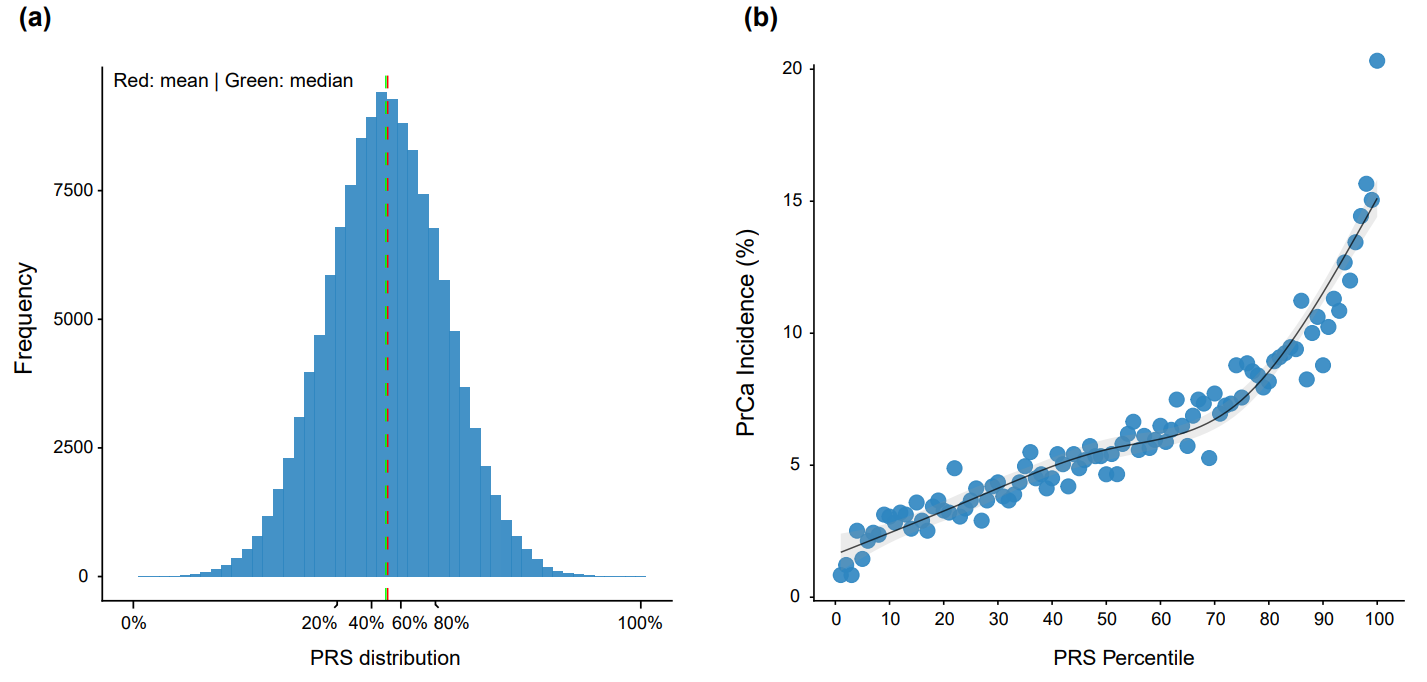


**(a)** Distribution of PRS for prostate cancer. Mean (red) and median (green) indicated. **(b)** Prostate cancer incidence by PRS percentile with natural cubic spline (4 df, black line; 95% CI shaded).

Monotonic dose-response relationship (*P* < 0.001).

PRS, polygenic risk score; PrCa, prostate cancer; PC, principal component; df, degrees of freedom; CI, confidence interval.

**Supplementary Figure 4.** Forest plots of risk of prostate cancer according to the genetic risk and diabetes mellitus status in the UK Biobank and Penn Medicine Biobank.

**
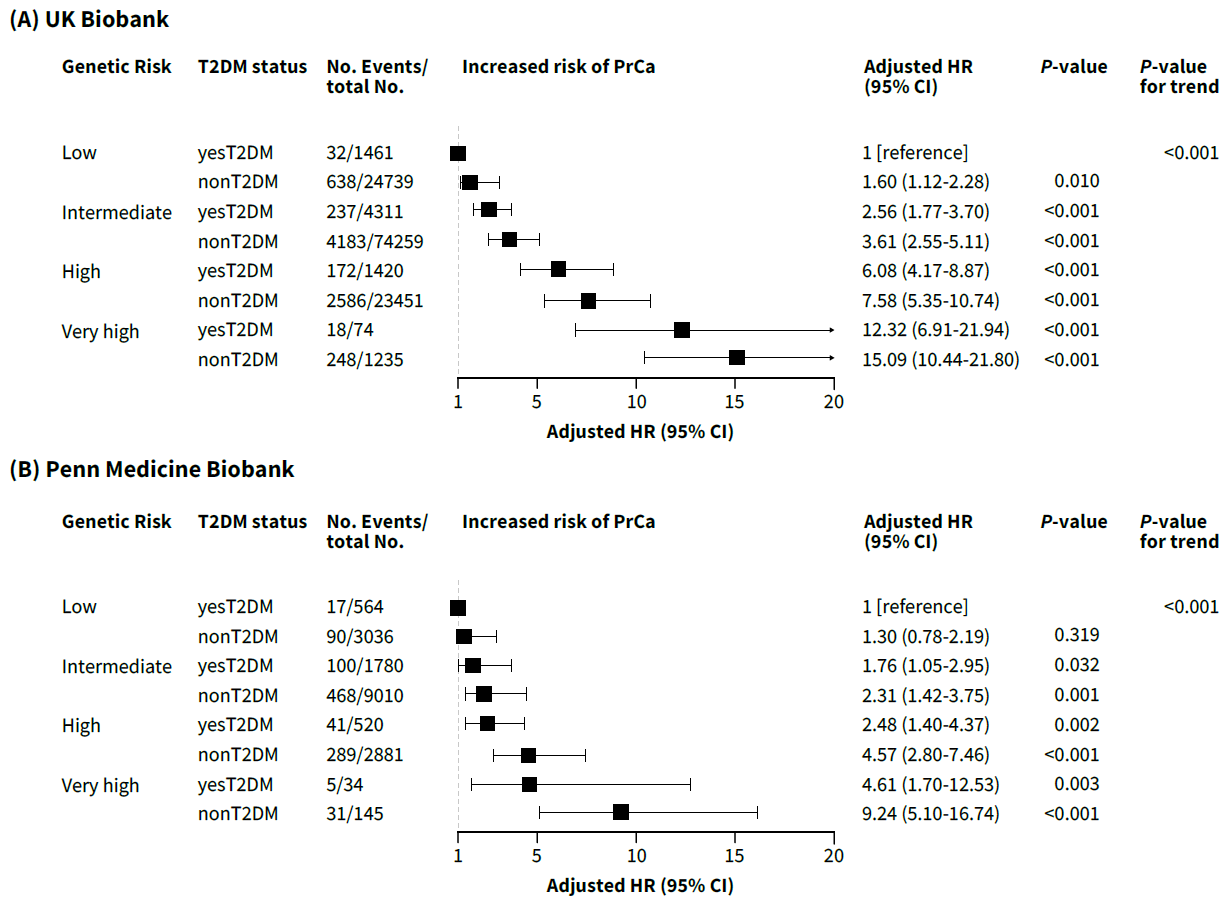
**

PrCa, prostate cancer; T2DM, type 2 diabetes mellitus; HR, hazard ratio; CI, confidence interval.

**Supplementary References**

1. Elliott P, Peakman TC. The UK Biobank sample handling and storage protocol for the collection, processing and archiving of human blood and urine. Int J Epidemiol. 2008;37(2):234-244.
2. Owen JM, Woods P. Designing and implementing a large-scale automated −80 degrees C archive. Int J Epidemiol. 2008;37 Suppl 1:i56-i61.
3. Sudlow C, Gallacher J, Allen N, et al. UK Biobank: an open access resource for identifying the causes of a wide range of complex diseases of middle and old age. PLoS Med. 2015;12(3):e1001779.
4. Perez-Cornago A, Key TJ, Allen NE, et al. Prospective investigation of risk factors for prostate cancer in the UK Biobank cohort study. Br J Cancer. 2017;117(10):1562-1571.
5. Howie BN, Donnelly P, Marchini J. A flexible and accurate genotype imputation method for the next generation of genome-wide association studies. PLoS Genet. 2009;5(6):e1000529.
6. O’Connell J, et al. Haplotype estimation for biobank-scale data sets. Nat Genet. 2016;48(7):817-820.
7. Fuchsberger C, Abecasis GR, Hinds DA. minimac2: faster genotype imputation. Bioinformatics. 2015;31(5):782-784.
8. Browning SR. Missing data imputation and haplotype phase inference for genome-wide association studies. Hum Genet. 2008;124(4):439-450.
9. Das S, et al. Next-generation genotype imputation service and methods. Nat Genet. 2016;48(10):1284-1287.
10. Marchini J, Abecasis G, Durbin R. A reference panel of 64,976 haplotypes for genotype imputation. Nat Genet. 2016;48(10):1279-1283.
11. 1000 Genomes Project Consortium. A global reference for human genetic variation. Nature. 2015;526(7571):68-74.
12. Tingley D, Yamamoto T, Hirose K, et al. Mediation: R package for causal mediation analysis. Journal of statistical software. 2014;59:1-38.
